# Rare Case of Peritonitis due to Ileal Perforation Secondary to Richter’s Type of Obturator Hernia

**DOI:** 10.7759/cureus.4289

**Published:** 2019-03-21

**Authors:** Zahid Ali Memon, Maria Aisha, Laila Tul Qadar, Rohan Kumar Ochani, Sarrah Ali Asghar

**Affiliations:** 1 Surgery, Dr. Ruth Km Pfau Civil Hospital, Karachi, PAK; 2 Internal Medicine, Dow University of Health Sciences, Karachi, PAK

**Keywords:** richter obturator hernia, small bowel obstruction, strangulation, perforation, peritonitis, computed tomography, howship-romberg sign, hannington kiff sign, surgery, comorbidity

## Abstract

Obturator hernia is an extremely rare condition accounting for almost 0.07%-1% of all abdominal wall hernias, usually occurring in the elderly and emaciated females with a history of previous abdominal surgery. The symptoms of this particular hernia are non-specific; therefore, a high index of clinical suspicion should always be made. This rare condition may lead to acute small intestinal obstruction. The pre-operative diagnosis is challenging and often misleading on occasions, especially in co-morbid cases. This leads to delayed diagnosis and surgical intervention, hence causing an increased morbidity and mortality rate. The computed tomography (CT) scan of the abdomen and pelvis is the gold standard for diagnosis. We present a case of an 80-year-old female, with known comorbid of hypertension, initially diagnosed as peritonitis and on further examination revealed strangulated obturator hernia with proximal perforation, that underwent lower midline laparotomy with resection of necrotic bowel, an end-to-end anastomosis, and repair of the defect by vicryl suture.

## Introduction

Obturator hernia is a rare type of abdominal wall hernia, accounting for 0.07%-1.0% of all hernias [1]. It is prevalent in elderly, emaciated, multiparous women usually with co-morbid, and develops more commonly on the right side. It frequently presents as mechanical small bowel obstruction caused by incarceration of the bowel into the obturator canal [2]. The incidence rate in Asians is higher than that of western people, as shown by a study conducted by Chung et al. which showed 16 diagnosed cases in a span of seven years [3]. The mortality rate of obturator hernia, which is highest among all abdominal wall hernias, is approximately 13%-40%.

The awareness of this condition is stressed due to infrequent incidence, non-specific symptoms, and inconclusive diagnostic imaging. The following reasons can make this condition a real diagnostic challenge. Early diagnosis and surgical intervention are imperative to reduce the risk of mortality and morbidity since delayed response can lead to strangulation of entrapped bowel loops. This case was initially diagnosed on computed tomography (CT) as peritonitis secondary to obturator hernia; later during laparotomy, it was found to be a Richter hernia.

## Case presentation

A frail, 80-year-old woman, known case of hypertension and chronic myeloid leukemia (CML) presented to the emergency department (ED) with a history of dull pain in the lower abdomen since the past four days. Along with this, she complained of absolute constipation, with three episodes of dark-colored non-projectile vomiting for four days. Her past medical history showed the presence of melena, constipation and gastroesophageal reflux disease (GERD), while her past surgical history revealed a laparoscopic cholecystectomy for cholelithiasis a long time ago. She is currently taking anti-hypertensive medications, hydroxyurea for CML and oral antacids to relieve abdominal pain when needed.

On examination (O/E), the patient was afebrile, comfortably lying on the bed and well oriented to time place and person with no signs of dehydration. Initial vitals included blood pressure (BP) of 150/100 mmHg, a regular pulse of 80 beats/min and a respiratory rate of 16 breaths/min. On inspection of the abdomen, she had diffuse abdominal distention, and while on palpation, she had mild diffuse tenderness at the right iliac fossa region, which was radiating towards left iliac fossa. Lastly, on auscultation, sluggish gut sounds were heard. No lymph nodes were palpable. Afterward, a nasogastric tube (NG) was inserted, which drained 200 ml of green-colored aspirate within 12 hours. The patient had negative Howship-Romberg sign and Hannington-Kiff sign. The hernial orifices were clinically normal, and the rectal examination was negative.

**Figure 1:**
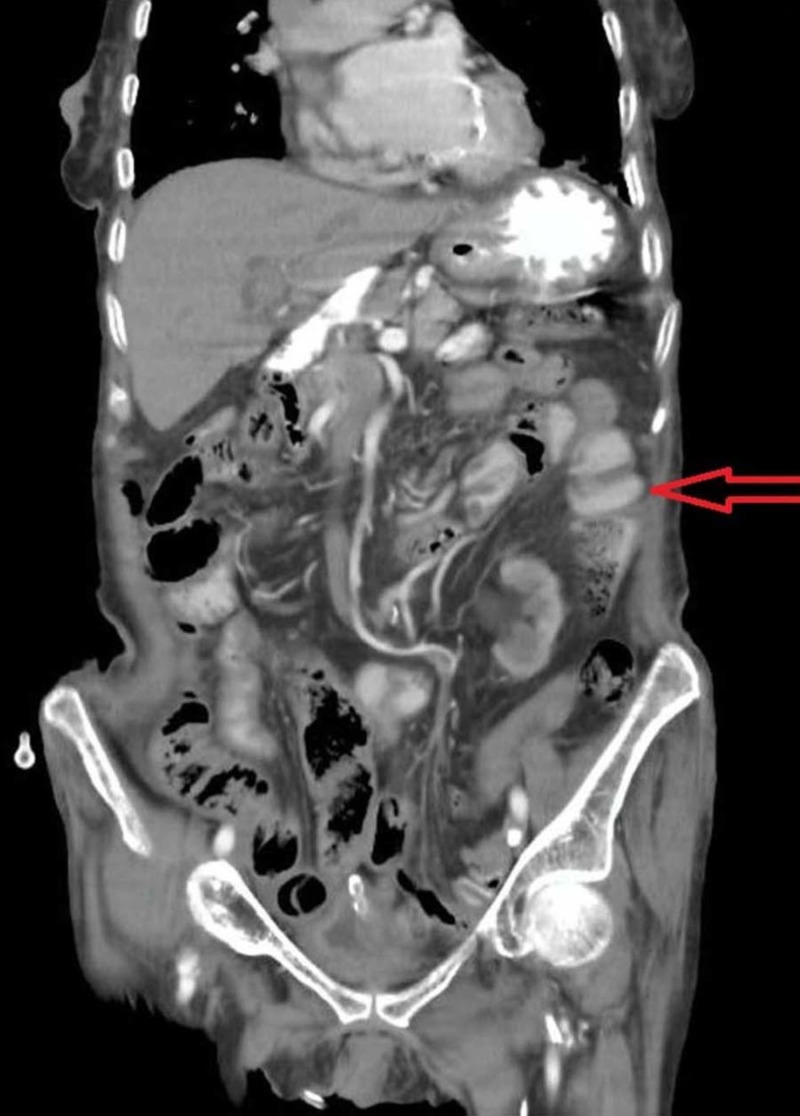
Computed tomography (CT) scan of the chest and abdomen revealing dilated bowel loops

Blood investigations revealed thrombocytosis and neutrophilic leukocytosis with a total leukocyte count (TLC) of 42.6x109/L. Serum electrolytes were abnormal which became normalized after adequate intake of fluid. The abdominal ultrasound reports showed dilated bowel loops and increased bowel gases. Left-sided small, simple renal cortical cysts were also noted. The chest and abdominal CT scans revealed dilated bowel loops and free gas under the diaphragm (Figure 1). The radiographs were reviewed and meticulously analyzed, and a right obturator hernia was identified.

The patient was then taken to the operating room for exploratory laparotomy. The procedure displayed 1 cm perforation in ileum on the anti-mesenteric side, which was 20 cm away from the ileocolic junction with obturator hernia of Richter’s type just distal to perforation. The loops of ileum were seen trapped between right pectineus and obturator externus muscle. This finding was pathognomonic of small bowel obstruction secondary to obturator hernia.

Hernial sac was reduced after performing adhesions and bowel decompression. A 10-cm resection of necrotic ileum was performed, then a two-layered primary end-to-end anastomosis was made using vicryl 3/0 followed by herniorrhaphy using a double-layer mesh for the closure of the obturator foramen. The midline incision was then closed in layers.

Finally, the patient developed metabolic acidosis on the fourth postoperative day which was immediately managed. Post-operative ultrasound of the abdomen and pelvis revealed right-sided mild pleural effusion and mild bilateral hydronephrosis. The patient had a satisfactory recovery and was doing well during the follow-up.

## Discussion

In the early 18th century, obturator hernia was first reported by Arnaud de Ronsil, and in the mid 19th century, it was first successfully repaired by Henry Ombre [4]. Obturator hernia occurs through an obturator canal, which is about 3 cm long formed by the two muscles, obturator externus, and internus. Obturator foramen is formed by the fusion of the ischial and pubic bones of the pelvis. It develops through a defect by separation of muscle fibers, first by, obturator internus, then obturator externus. The hernial sac mostly lies beneath pectineus muscle.

Obturator hernia is more common in elderly, thin, multiparous females. It is due to the loosening of pelvic floor muscles and absence of pre-peritoneal fat and lymphatic tissue, which usually envelops the obturator canal. The incidence ratio of male to female is 1:9 [5]. This female preponderance is due to a wider and shallower pelvis with a large obturator canal. Conditions which tend to increase intra-abdominal pressures, such as chronic obstructive pulmonary diseases, tend to be amongst the predisposing factors. It is relatively more common on the right side due to the presence of sigmoid colon on the left providing adequate protection to the obturator canal [6].

Hernial sac mainly contains the small bowel, which is 41%-100% of the cases is Richter’s type [5]. In Richter’s type, protrusion of peritoneum and strangulation or incarceration of only part of the lumen of the anti-mesenteric border of the intestine is seen to be into the obturator canal, due to a defect in the abdominal wall.

Primarily, the protrusion is of the terminal ileum into the hernial orifice, but any part of the intestinal tract, starting from the stomach to the colon, including the appendix may become incarcerated [7]. The hernial orifice in Richter’s type is firm and huge enough for the partial entrapment of bowel loops. Richter’s hernias tend to progress rapidly to gangrene, because the tight constricting ring may cause strangulation leading to gangrenous necrosis of the involved segment.

In Richter’s type hernia, due to partial obstruction, non-specific symptoms like nausea, vomiting and abdominal pain are less severe and may be overlooked, especially in cases of patients with co-morbid leading to a delayed diagnosis. This type of hernia tends to be diagnosed only after incarceration, due to the deep location, less frequent incidence, infrequent symptoms, and no palpable mass.

In about 15% of the cases, obturator hernia presents with a positive Howship-Romberg and Hannington-Kiff sign [1]. Howship-Romberg sign, which is considered pathognomonic, is pain felt along the medial aspect of thigh exacerbated by extension, abduction and internal rotation of hip due to compression of the obturator nerve. Another important diagnostic sign, the Hannington-Kiff sign, may be present, which is the absence of adductor reflex in the thigh in the presence of a positive patellar reflex, which is more specific for obturator hernia. Pectineus muscle usually covers the obturator sac so detecting a mass by external palpation is challenging; therefore, there is a little possibility of detecting palpable masses [8]. A high index of suspicion is present even in the absence of these signs.

The investigations include ultrasonography, CT, and magnetic resonance imaging (MRI). CT of the abdomen and pelvis is considered a gold standard for diagnosis and must be considered in elderly patients with non-specific small bowel obstruction. This condition is also diagnosed during emergency surgery for small bowel obstruction. The only possible treatment is surgical treatment, which can be through either laparotomy or laparoscopy, with repair of obturator canal either with omentum, mesh or sutures. The abdominal approach is often favored, although the laparoscopic approach has shown to have fewer complications [9]. The abdominal, low midline approach favors the identification of hernia and resection of gangrenous bowel if necessary. In the case of delayed surgical treatment, the strangulation rate can increase from 25% to 100% [10]. The preferred methods of herniorrhaphy include simple closure of the hernial defect with interrupted sutures or placement of a synthetic mesh as they are associated with the lowest complication rates [1]. The defect is not closed by the synthetic mesh in the case of perforation and gangrenous bowel, leading to significant complications. The postoperative mortality rate of these patients has been reported to be around 70%, due to delayed diagnosis, intestinal ischemia, and a high incident of perforation [11].

## Conclusions

Obturator hernia is a rare condition. It should be vigorously suspected as a cause of small bowel obstruction in thin, elderly females. In cases of great clinical suspicion of obturator hernia, a CT scan is essential to establish a pre-operative diagnosis. Often, diagnosis is delayed due to non-specific early symptoms, which can lead to perforation, peritonitis, and resection of gangrenous bowel, ultimately leading to significant post-operative mortality and morbidity. In a clinical setting, often obturator hernia is misdiagnosed as a femoral or inguinal hernia, which can lead to inappropriate management. Therefore, when accurately diagnosed, the only treatment is surgery, which is either through laparotomy or laparoscopy.
